# An ascites grading system for predicting the prognosis of gastric cancer with peritoneum dissemination

**DOI:** 10.1002/ags3.12386

**Published:** 2020-08-16

**Authors:** Michitaka Honda, Hidetaka Kawamura, Hiroshi Kobayashi, Koichi Takiguchi, Atsushi Muto, Shigeru Yamazaki, Yasushi Teranishi, Satoru Shiraso, Koji Kono, Soshi Hori, Takahiro Kamiga, Toshiyasu Iwao, Naoyuki Yamashita

**Affiliations:** ^1^ Department of Minimally Invasive Surgical and Medical Oncology Fukushima Medical University Fukushima Japan; ^2^ Department of Surgery Southern TOHOKU General Hospital Koriyama Japan; ^3^ Department of Surgery The Takeda Healthcare Foundation Takeda General Hospital Aizuwakamatsu Japan; ^4^ Department of Surgery Fukushima Rosai Hospital Iwaki Japan; ^5^ Department of Surgery Ohta Nishinouchi Hospital Koriyama Japan; ^6^ Department of Surgery Iwaki City Medical Center Iwaki Japan; ^7^ Department of Gastrointestinal Tract Surgery Fukushima Medical University Fukushima Japan; ^8^ Department of Surgery Shirakawa Kosei General Hospital Shirakawa Japan; ^9^ Department of Internal Medicine Aidu Chuo Hospital Aizuwakamatsu Japan; ^10^ Department of Surgery Tsuboi Hospital Koriyama Japan

**Keywords:** ascites, gastric cancer, peritoneal dissemination, prognosis

## Abstract

**Aim:**

Gastric cancer with peritoneum dissemination is intractable with surgical resection. The evaluation of the degree of dissemination using computed tomography (CT) is difficult. We focused on the amount of ascites based on CT findings and established a scaling system to predict these patients’ prognoses.

**Methods:**

We extracted individual data from a population‐based cohort. Patients diagnosed with histologically proven gastric adenocarcinoma with peritoneum dissemination were enrolled. Two raters evaluated the CT images and determined the grade of ascites in each patient: grade 0 indicated no ascites in all slices; grade 1 indicated ascites detected only in the upper or lower abdominal cavity; grade 2 indicated ascites detected in both the upper and lower abdominal cavities; and grade 3 indicated ascites extending continuously from the pelvic cavity to the upper abdominal cavity. We evaluated the relationship between the ascites grade and survival time. After adjusting for other clinical factors, we calculated hazard ratios of each ascites grade.

**Results:**

A total of 718 patients were enrolled. The number of patients with grades 0, 1, 2, and 3 were 303, 223, 94, and 98, respectively. The median overall survival times were 16.0, 8.7, 5.4, and 3.0 months for ascites on CT grades 0, 1, 2, and 3, respectively (*P* < .001). The adjusted hazard ratios for the survival time were 1.74 (1.33‐2.26, *P* < .001), 3.20 (2.25‐4.57, *P* < .001), and 4.76 (3.16‐7.17, *P* < .001) for grades 1, 2, and 3, respectively.

**Conclusion:**

We established a new grading system of pretreatment ascites to better predict the prognosis of gastric cancer.

## Graphical abstract

This study showed the clinical validity of novel grading system for pretreatment ascites that can predict the prognosis of gastric cancer patients with peritoneal dissemination.

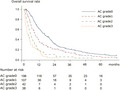

## 1 INTRODUCTION

Peritoneal dissemination (PD) is one of most common metastatic patterns of gastric cancer (GC).^1^, ^2^ The prognosis of GC patients with PD remains poor, despite recent advances in anticancer agent development.^3^ The progression of PD is often accompanied by various symptoms, including abdominal pain, fullness, and vomiting caused by intestinal obstruction or massive ascites.

Surgeons often detect PD during surgery for GC patients scheduled for radical resection.^4^ As imaging examinations cannot detect the small nodules characteristic of PD,^5^ laparotomy or staging laparoscopy is sometimes required to observe the intra‐abdominal cavity.^6^ Serious problems hampering the treatment of GC patients with PD include difficulty assessing the degree of PD and predicting the prognosis. Jacquet and Sugarbaker previously proposed a scaling method of PD for all kinds of malignant disease,^7^ and Fujimura et al reported a modified version of this scaling system for GC patients with PD to predict patients’ prognoses.^8^ These methods are based on macroscopic findings; however, a scaling system without invasiveness is also required from a physician's perspective, as many patients with PD do not receive surgery.

Previous reports have described several prognostic factors in patients with PD, such as the nutritional condition,^3^, ^9^, ^10^ performance status,^3^ tumor markers,^11^ and the presence of ascites.^3^, ^4^, ^12^, ^13^ However, which of these is most useful for estimating the degree of ascites as a surrogate marker of PD in patients with GC is unclear.

In the present study, we focused on the amount of ascites at the initial diagnosis and the scaling system based on computed tomography (CT) findings. We investigated the relationship between the amount of ascites on CT and the prognosis of GC with PD using our established large‐scale cohort in an effort to establish a physician‐friendly prediction tool involving the assessment of ascites to predict the prognosis of GC with PD.

## 2 METHODS

### 2.1 Study design and cohort development

The present study was a population‐based historical cohort study. All nine designated hospitals for cancer treatment in Fukushima Prefecture participated in this study. First, we listed the patients with stage IV GC using hospital‐based cancer registries. We then obtained these patients’ individual data along with additional information, including the Charlson's comorbidity index,^14^ symptoms, cTNM stage,^15^ CT findings, laboratory data, and treatment. Finally, we merged the datasets from each participating institute after anonymizing the information.

We enrolled patients in this study according to the criteria described below. Patients were diagnosed with GC (ICD‐10, C16.0‐16.9) with synchronous PD and histologically proven adenocarcinoma from a primary lesion from 2008 to 2015. Patients who did not undergo abdominopelvic CT before any treatment, who were lost to follow‐up, who had remnant GC after previous gastrectomy, who had other synchronous malignancies, or who had a medical history of liver cirrhosis, congestive heart failure, or use of diuretics were excluded. Investigators confirmed that the helical CT scans included the area from the diaphragm to the symphysis pubis in 5‐ to 10‐mm‐thick transverse sections at the same intervals.

This study was conducted in accordance with the Declaration of Helsinki and all of the applicable local laws and regulations. Approval for the protocol was obtained from the institutional review boards of all of the participating hospitals.

### 2.2 The diagnosis of PD and grading of ascites

We reviewed medical records of all patients in this cohort and diagnosed the PD according to the findings as follows: having visible nodules near the primary lesion on CT images, positive cytology from aspirated ascites or lavage cytology, or histologically proven disseminated lesions during/after surgery, including staging laparoscopy.

In addition, two gastrointestinal surgeons (MH and HK), who were blinded to the survival outcome, reviewed abdominal CT before initial treatment and classified patients into four categories depending on the amount of ascites detected at the initial diagnosis. We established the ascites on CT (AC) grading system by referencing the methods used in published clinical trials^16^, ^17^: grade 0 indicated no ascites in all slices; grade 1 indicated ascites detected only in the upper or lower abdominal cavity; grade 2 indicated ascites detected in both the upper and lower abdominal cavities; and grade 3 indicated ascites extending continuously from the pelvic cavity to the upper abdominal cavity.

### 2.3 Outcomes and statistical analyses

The primary outcome was the adjusted hazard ratio (HR) of each AC grade for the overall survival (OS). To compare the OS by AC grade, we evaluated the descriptive statistics and isolated potential confounding factors. After adjusting for the age, sex, nutritional condition, comorbidities, tumor markers, and treatment with or without chemotherapy as confounding factors, we calculated the HR and 95% confidence interval (95% CI) of each AC grade using the Cox proportional hazard model. We also calculated the adjusted HR in patients with no metastatic lesions other than PD as subclass analysis. In addition, we evaluated the survival curve for these patients using the Kaplan‐Meier method for each AC grade and performed the Wilcoxon test.

Secondary outcomes were nutritional indicators, tumor markers, proportion of chemotherapy or gastrectomy, and incidence of synchronous liver metastasis in each AC grade. The descriptive statistics were evaluated, and as necessary, continuous variables were compared using Student's t‐test and categorical variables by Fisher's exact test. All statistical tests were two‐sided, and *P* values of .05 or less were considered to indicate statistical significance.

## 3 RESULTS

**Figure 1:**
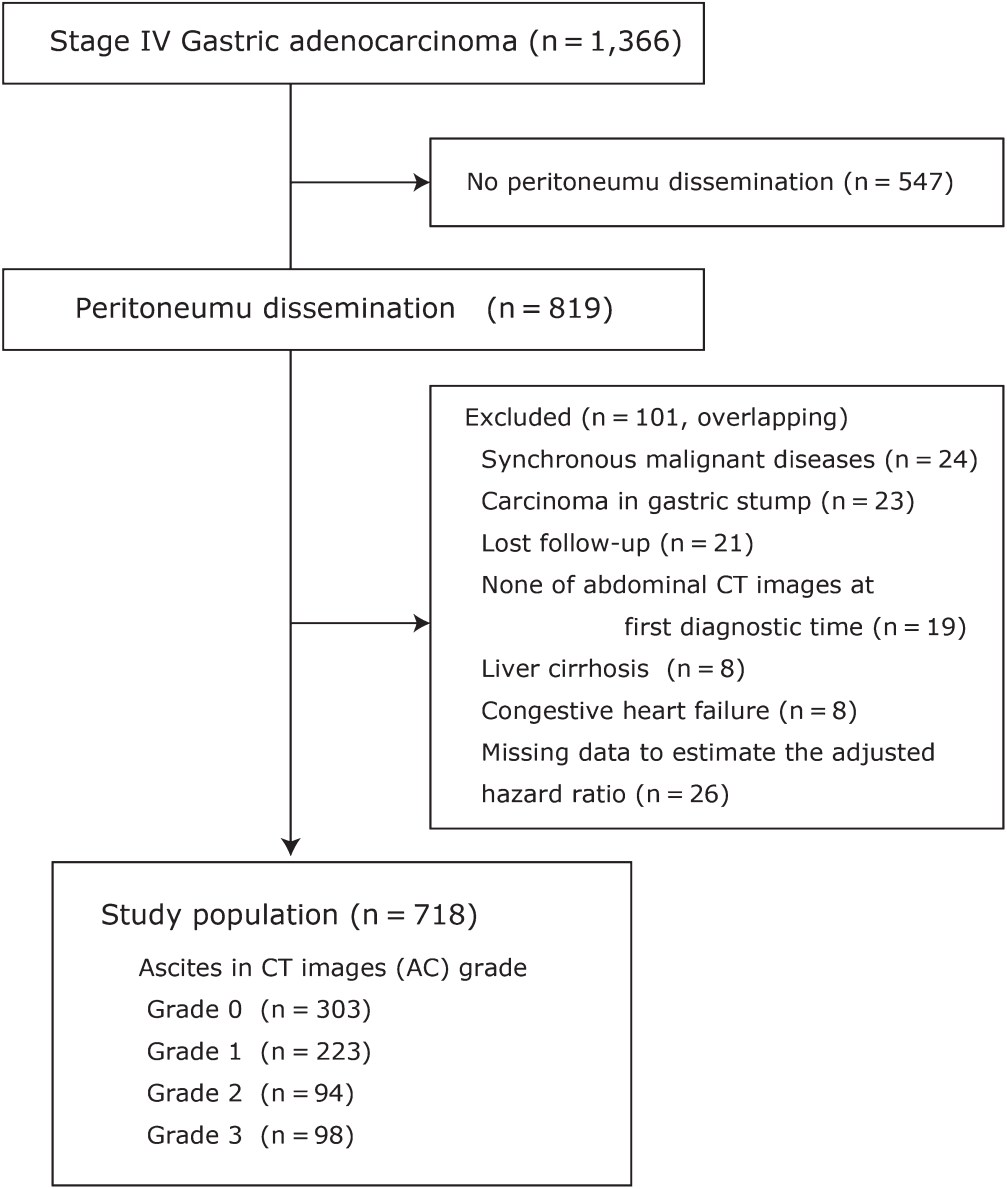
Patients’ enrollment. All of 718 patients were extracted from the population‐based cohort of stage IV gastric cancer

Figure 1 shows the patient enrollment flow. A total of 1366 patients with stage IV GC were identified from the cohort databases, including 819 patients diagnosed with PD; ultimately, 718 patients were enrolled in this study. Table 1 shows the patients’ characteristics. The number of patients with AC grades of 0, 1, 2, and 3 was 303, 223, 94, and 98, respectively.

**Table 1:** Patients' characteristics

	Total N = 718	(%)
Age		
Median [range]	70 [23‐98]	
Sex		
Male	468	65.2
Female	250	34.8
Charlson's comorbidity index		
0‐2	677	94.3
3<	41	5.7
Body mass index		
Mean (SD)	20.1 (8.15)	
Clinical T		
<T3	56	7.8
>T4a	662	92.2
Clinical N		
cN0	131	18.2
cN+	579	80.6
Histological findings		
Well to mode	198	27.6
Poor sig muc	411	57.2
Mixed	80	11.1
Diagnosis of Peritoneal dissemination		
CT findings	318	44.3
Macroscopic findings	164	22.8
Cytology	126	17.5
Other metastatic organs		
None (only peritoneum)	386	53.8
Lymph node	214	29.8
Liver	181	25.2
Lung	27	3.8
Bone	15	2.1
Others	29	4.0
AC grade		
0	303	42.2
1	223	31.1
2	94	13.1
3	98	13.6

Abbreviations: AC grade, ascites on computed tomography grade; SD, Standard deviation.

### 3.1 Adjusted HRs and overall survival curves

**Figure 2:**
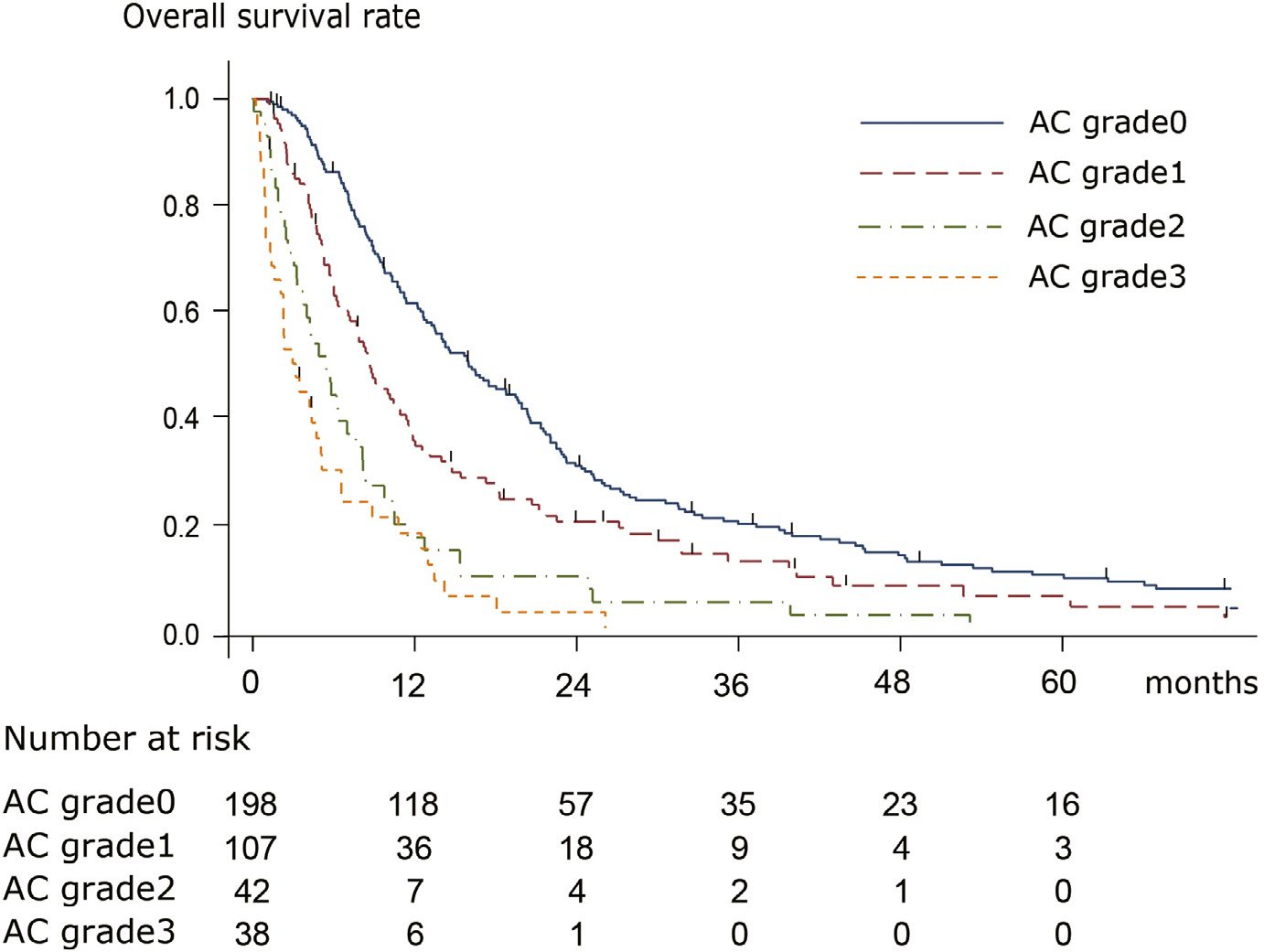
Survival curve of each ascites grade. Overall survival curves using for each AC grade using Kaplan‐Meier's methods

The median OS was 7.8 and 10.5 months in all patients (n = 718) and patients without other metastatic lesions (n = 386), respectively. Table 2 shows the adjusted HRs for all patients. The adjusted HRs (95% CI) were 1.74 (1.33‐2.26), 3.20 (2.25‐4.57), and 4.76 (3.16‐7.17) for AC grades 1, 2, and 3, respectively. Table 3 also shows the adjusted HRs in patients with no metastatic lesions other than PD. Figure 2 shows the OS rate and at‐risk population of each AC grade among patients who did not have other metastatic lesions (*P* < .001). The median OS values were 16.0, 8.7, 5.4, and 3.0 months for AC grades 0, 1, 2, and 3, respectively (*P* < .001).

**Table 2:** Adjusted hazard ratio for overall survival in all patients

Variables	Rerefence	HR	*P* value	95% CI
AC Grade 1	Grade 0	1.74	<.001	1.33‐2.26
AC Grade 2	Grade 0	3.20	<.001	2.25‐4.57
AC Grade 3	Grade 0	4.76	<.001	3.16‐7.17
Age (>75)	<75	1.00	.649	0.99‐1.01
Sex (Female)	Male	0.98	.889	0.78‐1.24
Chemotherapy	No chemotherapy	0.44	<.001	0.33‐0.59
Liver metastasis	None	1.65	<.001	1.39‐1.96
Charlson's score > 3	<2	1.13	.062	0.99‐1.29
Albumin level (<3.0 g/dL)	>3.0 g/dL	1.42	.042	1.01‐1.99
BMI (<18.5 kg/m^2^)	>18.5 kg/m^2^	0.88	.356	0.68‐1.15
Lymphocyte (<1000/µL)	>1000/µL	1.28	.141	0.92‐1.78
CEA level (>5.0 ng/mL)	<5.0 ng/mL	1.17	.220	0.91‐1.50
CA19‐9 level (>37.0 ng/mL)	>37.0 ng/mL	1.23	.086	0.97‐1.57

Abbreviations: AC Grade; ascites on computed tomography grade, BMI; body mass index; carbohydrate antigen 19‐9; carcinoembryonic antigen, CA19‐9; CEA.

**Table 3:** Adjusted hazard ratio in patients with no metastatic lesions other than PD

Variables	Rerefence	HR	*P* value	95% CI
AC Grade 1	Grade 0	1.60	.001	1.22‐2.10
AC Grade 2	Grade 0	2.45	<.001	1.62‐3.69
AC Grade 3	Grade 0	3.96	<.001	2.34‐5.97
Age (>75)	<75	1.00	.711	0.99‐1.01
Sex (Female)	Male	0.99	.927	0.78‐1.25
Chemotherapy	No chemotherapy	0.42	<.001	0.31‐0.55
Charlson's score > 3	<2	1.10	.155	0.96‐1.26
Albumin level (<3.0 g/dL)	>3.0 g/dL	0.87	.124	0.73‐1.04
BMI (<18.5 kg/m^2^)	>18.5 kg/m^2^	0.88	.338	0.67‐1.14
Lymphocyte (<1000/µL)	>1000/µL	1.21	.262	0.87‐1.69
CEA level (>5.0 ng/mL)	<5.0 ng/mL	1.17	.215	0.91‐1.50
CA19‐9 level (>37.0 ng/mL)	>37.0 ng/mL	1.26	.058	0.99‐1.61

Abbreviations: AC Grade, ascites on computed tomography grade; BMI, body mass index; CA19‐9, carbohydrate antigen 19‐9; CEA, carcinoembryonic antigen.

### 3.2 Secondary outcomes

Table 4 shows the relationship between the AC grade and malnutrition or treatment as the secondary endpoints.

**Table 4:** Ascites grade and other potential risk factors

AC grade	Grade 0		Grade 1		Grade 2		Grade 3		*P* value
	n = 303	%	n = 223	%	n = 94	%	n = 98	%	
Chemotherapy									
First line	216	71.3	168	75.3	54	57.4	52	53.1	<.001
Second line	105	34.7	82	36.8	16	17.0	19	19.4	<.001
Third line	62	20.5	33	14.8	6	6.4	10	10.2	.003
Surgery									
Primary resection	201	66.3	66	29.6	7	7.4	3	3.1	<.001
Nutrition									
Body mass index < 17.0	64	21.1	44	19.7	14	14.9	12	12.2	.178
Albumin < 3.0 g/dL	26	8.6	41	18.4	22	23.4	28	28.6	<.001
Lymphocyte < 1000/μL	27	8.9	40	17.9	19	20.2	22	22.4	.001
Tumor marker									
CEA (>5.0 ng/mL)	131	43.2	96	43.0	49	52.1	52	53.1	.166
CA19‐9 (>37.0 ng/mL)	120	39.6	102	45.7	47	50.0	45	45.9	.245
Liver metastasis	62	20.5	54	24.2	31	33.0	34	34.7	.010

Abbreviations: AC grade, ascites on computed tomography grade; CA19‐9, carbohydrate antigen 19‐9; CEA, carcinoembryonic antigen.

## 4 DISCUSSION

Our study proposed the AC grading system, which is a new scale of ascites using abdominopelvic CT as a prognostic tool in GC patients with PD. Patients with a larger amount of ascites generally have a poorer nutritional condition and higher proportion of other metastatic organs than those with less ascites. We confirmed in the present study that the AC grade indicated a linear increase in the HR for the OS in each grade after adjusting for these factors.

A method for measuring the degree of PD from any malignancy has yet to be established. Even if suspicious findings are not detected on CT, surgeons sometimes find PD during surgery for patients with GC. Indeed, staging laparoscopy detected PD in 45% to 47% of patients with diffuse filtration‐type or large infiltrative ulcer‐type GC.^18^, ^19^ The Japanese Research Society for Gastric Cancer originally proposed that PD be classified into four grades by the anatomical spread of lesions (P0, P1, P2, and P3); however, the relationship between the grades and prognosis was never determined, and this grading system was abolished in a later classification.^20^ Sugarbaker et al developed a scoring method for evaluating the volume of PD for all malignant diseases; their method divided the peritoneal or mesentery surface into 13 regions and combined to four volume scoring.^7^ In addition, Fujimura et al reported that their modified PD scaling system was useful for predicting the prognosis of patients with GC, although they showed a significant difference in the survival time between just two categories: grade I and II to IV.^8^ Several other studies have further identified specific proteins or mRNA in ascites or lavage fluid that may be candidate biomarkers for predicting the prognosis.^21^, ^22^, ^23^ However, many PD patients receive treatment without ever undergoing surgery, as the effectiveness of debulking surgery has been denied by the previous reported phase III clinical trial.^24^ The development of a simple and low‐invasive prediction tool would therefore be quite useful for both patients and physicians.

We established our AC grading system by referencing the method of assessing ascites in a previous phase III clinical trial evaluating combination chemotherapy of 5‐fluorouracil, l‐leucovorin, and paclitaxel.^16^ Aside from our grading system, a number of other scaling systems have also been reported, including ones based on physical findings or CT images. For example, Oriuchi et al developed an equation for approximating the amount of ascites by measuring the thickness at five points on abdominopelvic CT images.^25^ Although the AC grading system is not able to estimate the volume of ascites directly, the easy evaluation, adjusting other prognostic factors, and strong OS discrimination suggest that this would be a clinically relevant tool for physicians treating GC.

Other strong points of this study are its high external validity for the targeted population and the accuracy of the measurements. We established a large, population‐based cohort in a medical field, and fixed investigators reviewed CT images of all cases and decided the AC grades based on predetermined criteria.

One of the limitations in this study is that we did not evaluate the indication of chemotherapy and therapeutic response. Although most of the physicians prescribed chemotherapy according to Japanese guidelines in participating institutes, the decisions of regimen or dosage were not completely same. In addition, the trends in ascites, clinical symptoms, and serum tumor marker levels may be useful for deciding to change a regimen to a later line. We are planning a further evaluation using the trend in the ascites volume during chemotherapy. Another limitation was the accuracy of the measurement modality. To minimize information bias, two fixed raters evaluated AC grade in this study; however, the CT parameters, such as the slice range setting or use of contrast medium, were not unified due to the multicenter retrospective nature.

In conclusion, we established the clinical meaning of the new grading system for pretreatment ascites that can predict the prognosis of GC patients with PD.

## DISCLOSURE

Conflict of Interest: Authors declare no conflict of interests for this article.
